# Imaging the injured beating heart intravitally and the vasculoprotection afforded by haematopoietic stem cells

**DOI:** 10.1093/cvr/cvz118

**Published:** 2019-05-07

**Authors:** Dean P J Kavanagh, Adam B Lokman, Georgiana Neag, Abigail Colley, Neena Kalia

**Affiliations:** Institute of Cardiovascular Sciences, College of Medical and Dental Sciences, University of Birmingham, Birmingham, UK

**Keywords:** Coronary microcirculation, Ischaemia–reperfusion injury, Intravital microscopy

## Abstract

**Aims:**

Adequate microcirculatory perfusion, and not just opening of occluded arteries, is critical to salvage heart tissue following myocardial infarction. However, the degree of microvascular perfusion taking place is not known, limited primarily by an inability to directly image coronary microcirculation in a beating heart *in vivo*. Haematopoietic stem/progenitor cells (HSPCs) offer a potential therapy but little is known about their homing dynamics at a cellular level and whether they protect coronary microvessels. This study used intravital microscopy to image the anaesthetized mouse beating heart microcirculation following stabilization.

**Methods and results:**

A 3D-printed stabilizer was attached to the ischaemia–reperfusion injured (IRI) beating heart. The kinetics of neutrophil, platelet and HSPC recruitment, as well as functional capillary density (FCD), was imaged post-reperfusion. Laser speckle contrast imaging (LSCI) was used for the first time to monitor ventricular blood flow in beating hearts. Sustained hyperaemic responses were measured throughout reperfusion, initially indicating adequate flow resumption. Intravital microscopy confirmed large vessel perfusion but demonstrated poor transmission of flow to downstream coronary microvessels. Significant neutrophil adhesion and microthrombus formation occurred within capillaries with the latter occluding them, resulting in patchy perfusion and reduced FCD. Interestingly, ‘patrolling’ neutrophils were also observed in capillaries. Haematopoietic stem/progenitor cells readily trafficked through the heart but local retention was poor. Despite this, remarkable anti-thromboinflammatory effects were observed, consequently improving microvascular perfusion.

**Conclusion:**

We present a novel approach for imaging multiple microcirculatory perturbations in the beating heart with LSCI assessment of blood flow. Despite deceptive hyperaemic responses, increased microcirculatory flow heterogeneity was seen, with non-perfused areas interspersed with perfused areas. Microthrombi, rather than neutrophils, appeared to be the major causative factor. We further applied this technique to demonstrate local stem cell presence is not a pre-requisite to confer vasculoprotection. This is the first detailed *in vivo* characterization of coronary microcirculatory responses post-reperfusion injury.

## 1. Introduction

Treatment of myocardial infarction (MI) focuses on rapidly re-establishing reperfusion following blockage in one or more of the coronary arteries. Despite successful thrombolytic and primary percutaneous coronary interventions (PCIs), a significant proportion of patients still incur muscle damage and proceed to develop heart failure.^1^^,^^2^ Indeed, restoration of normal epicardial blood vessel flow following PCI, but with sub-optimal myocardial perfusion, can be observed in as high as 50% of patients.^3^^,^^4^ This is strongly associated with larger infarcts, reduced ventricular function and overall worse outcomes than in patients with full perfusion recovery.^5^^,^^6^ It is likely that in the absence of overt atherosclerotic disease or occlusions within larger and clinically visible conduit vessels, tissue damage occurs subsequent to inadequate coronary microcirculatory perfusion. Indeed, in recent years, cardiovascular magnetic resonance (CMR) imaging has provided new insights into the significant impact microvascular obstruction and intramyocardial haemorrhage has on clinical outcomes.^7^ Furthermore, progressive loss of distal perfusion at the tissue level and neutrophil accumulation has been demonstrated in patients post-MI using positron-emission tomography (PET) scanning.^8^ However, current clinical imaging tools such as X-ray angiography, CMR, and PET cannot spatially resolve blood vessels less than 200 μm in diameter and so are unable to image small capillaries nor the blood cells within them at a cellular level.

An inability to directly image microvessels of the heart has led to cardiologists focusing their efforts on improving flow within the angiographically visible part of the coronary circulation. This has prevented a complete identification of the role of the microcirculation in ischaemic cardiovascular disease. The microcirculation comprises the functional ‘business’ end of the circulation and, in the heart, is responsible for 75% of myocardial blood flow. The significant oxygen demand of the heart is provided by an extensive dense network of capillaries running between all muscle fibres providing almost every fibre with its own capillary. The microvasculature controls total coronary resistance and is thus critical in regulating myocardial blood flow. Moreover, it is highly responsive to, and a vital participant in, the inflammatory response. Dysfunction of the coronary microvasculature has recently emerged as a potentially important mechanism contributing to MI with important prognostic implications. Indeed, an increasing number of publications have emphasized the need to focus research interests into studying the coronary microcirculation from both a basic and clinical perspective and prevent it remaining as a research ‘black box’.^9–12^ The importance of the microcirculation in heart disease is further exemplified in conditions such as microvascular angina (cardiac syndrome X) and in MI with non-obstructive coronary arteries (MINOCA) where patients present with the clinical features of an acute MI but without angiographic evidence of obstructive coronary arteries.^12–14^

Currently, little is known about the full range of microcirculatory responses to MI *in vivo*, limited primarily by an inability to directly image these events in a beating heart. Functional readouts, such as blood flow and pressure, can be determined clinically using a Doppler flow wire inserted into the coronary artery or assessed using thrombolysis in MI (TIMI) flow or myocardial blush. However, these assessments are mainly of larger vessels and actual visualization of coronary microcirculation, let alone any detrimental events taking place within them, remains impossible with current clinical imaging tools. Experimental knowledge to date has commonly been obtained histologically, but these static snapshots provide no indication of the real-time kinetics of inflammatory cell recruitment—a dynamic process—in the presence of pathophysiological flow. Tissue sections also do not offer any information on microvessel integrity and perfusion. Consequently, experts have recently suggested creating an international ‘coronary microcirculatory observatory’, a virtual facility utilizing imaging techniques such as intravital microscopy (IVM) to properly interrogate the coronary microcirculation in the beating heart of living animals.^11^

However, real-time microscopic imaging of the beating heart has been very challenging as motion compromises spatial and temporal resolution and therefore requires ‘stabilization’ of the heart—this in itself is technically very difficult. We have developed a stabilization method that sufficiently reduces motion in a small region of the beating left ventricle to permit intravital imaging of the coronary microcirculation. Li *et al.*^15^ have used a somewhat similar approach to monitor dynamic neutrophil events in a donor beating heart transplanted in the neck of a recipient. However, we present further novel data illustrating the extensive nature of neutrophil and platelet adhesion that occurs in the microcirculation following myocardial ischaemia–reperfusion injury (IRI) in a native heart *in situ*. In particular, we show platelet microthrombi occluding significant lengths of the capillaries and the considerable detrimental impact of these occlusions on functional capillary density (FCD). Furthermore, we combine IVM with full field laser speckle contrast microscopy to correlate microvascular events with whole beating heart blood perfusion. Importantly, we show poor microcirculatory perfusion is observed intravitally despite an overall reactive hyperaemic response detected using laser speckle imaging. Increased clinical recognition of the importance of the coronary microcirculation has meant identifying strategies to improve detrimental perturbations within it has also gained recent attention. To this effect, we also investigated the homing of systemically delivered exogenous bone marrow (BM)-derived haematopoietic stem/progenitor cells (HSPCs) to the injured heart. This is the first study to directly image, at a cellular level, the myocardial homing and trafficking of any exogenous stem cell type *in vivo*. We demonstrate their remarkable ability to confer vasculoprotection, and have elucidated potential therapeutic mechanisms of action, which occurs despite limited interactions with the coronary microcirculation and poor myocardial retention.

## 2. Methods

### 2.1 Myocardial ischaemia–reperfusion injury

Experiments were conducted on male C57BL/6 mice (8–12 weeks) in accordance with the Animals (Scientific Procedures) Act of 1986 (ASPA) which enforces Directive 2010/63/EU of the European Parliament on the protection of animals used for scientific purposes (Project licence: P552D4447). We have previously reported the microcirculatory response to IRI, and HSPC trafficking, within the liver, gut, and kidney of male mice. In order to make meaningful comparisons between these events in different vascular beds, the current work was also performed in male mice. Anaesthesia was induced by I.P. administration of ketamine hydrochloride (100 mg/kg) and medetomidine hydrochloride (100 mg/kg) and maintained as required via intra-arterial administration. Mice were intubated and ventilated with medical oxygen via a MiniVent rodent ventilator (stroke volume: 220 μL, respiratory rate: 130 pm; Biochrom Ltd. Harvard Apparatus, UK). The carotid artery was cannulated to facilitate infusion of fluorescently labelled cells, antibodies, dyes and maintenance anaesthetic. Ischaemia–reperfusion injury was induced by ligating the left anterior descending (LAD) artery for 45 min with reperfusion initiated for 2 h by removal of the suture.

### 2.2 Haematopoietic stem/progenitor cells—HPC-7 cells

Intravital studies monitoring HSPC trafficking have been limited due to difficulties in isolating sufficient numbers for *in vivo* experimentation. Therefore, an immortalized HSPC line, HPC-7, was used. These cells have characteristics similar to primary cells including surface expression of common murine HSC markers (c-kit^+^, CD34^−^, Lin^−^) and surface adhesion molecules and are also able to reconstitute haematopoiesis when injected into lethally irradiated hosts.^16^ We have previously used HPC-7s to model hepatic and intestinal HSPC recruitment.^17–20^ To image HPC-7s, they were labelled with 5 µM carboxyfluorescein diacetate succinimidyl ester (CFDA-SE; Life Technologies, UK) as previously described.^17–20^ To assess whether their adhesion could be enhanced in the heart *in vivo*, HPC-7s were pre-treated with hydrogen peroxide (H_2_O_2_, 100 μM; 1 h) prior to infusion. This strategy has previously been used by us to successfully enhance HSPC retention in the gut.^18^

### 2.3 Characterizing injury on coronary endothelium and myocytes using flow cytometry

Flow cytometry on digested heart cells was developed to determine whether coronary microvasculature was more susceptible to IRI than myocytes and whether HSPCs could modify inflammatory and oxidative injury. Ventricles were mechanically digested using collagenase type-I dispersion solution (0.1%; Wako Chemicals, Japan) and strained through a 70 μm filter into media (DMEM, 10% FCS, 1% penicillin/streptomycin; Sigma). Cells were isolated by centrifugation, fixed, permeablized with 70% ethanol and then re-suspended in blocking agent (rat anti-mouse CD16/32 antibody; BioLegend). Washed cells were incubated with antibodies as required: PE-conjugated rat anti-mouse CD31 (dilution 1:50; 390; ThermoFisher Scientific), PE-conjugated rat IgG2a control (dilution 1:50; eBR2a; ThermoFisher Scientific), PE-conjugated human anti-mouse/human/rat cardiac Troponin-T (cTnT; dilution 1:10; REA400; Miltenyi Biotec), Alexa 488 conjugated rat anti-mouse ICAM-1 (1:50), PE-Cy7 anti-mouse VCAM-1 (1:50), PE-conjugated human REA control (dilution 1:10; REA293; Miltenyi Biotec), purified polyclonal goat anti-8-OHdG (dilution 1:500; Abcam), purified goat IgG control (dilution 1:500, Abcam). Data analysis was performed on washed cells using FACSCalibur (BD Biosciences). Adhesion molecule expression and oxidative damage on cTnT^+^ myocytes and CD31^+^ ECs was quantitated in 30 000 cellular events and expressed as mean fluorescence intensity.

### 2.4 8-OHdG and dihydroethidium staining of vena cava endothelial cells *in vitro*

The ability of HSPCs to modify oxidative damage or limit the generation of superoxide species within cardiovascular endothelial cells (EC) following ROS challenge was assessed *in vitro*. Immortomouse-derived vena cava ECs (VCEC)^17^ were grown to confluence and then exposed to H_2_O_2_ (100 μM) to oxidatively damage them for 24 or 72 h in the presence or absence of 5 × 10^4^ HSPCs. To assess oxidative damage, ethanol fixed and permeabilized VCECs were incubated with a polyclonal goat anti-mouse 8-OHdG antibody (dilution 1:500) followed by Alexa 488-conjugated donkey anti-goat IgG antibody (dilution 1:250). Dihydroethidium (DHE; 500 μL; 10 μM) was added to detect superoxide generation. Cells were imaged using EVOS and integrated density was calculated for 8-OHdG and DHE.

### 2.5 Intravital imaging of the cardiac microcirculation

To facilitate intravital visualization of epicardial microcirculation in the anaesthetized mouse beating heart, we modified a stabilizer previously described by Lee *et al*.^21^ A small 3D-printed ring, with internal and external diameters of 2.25 mm and 4 mm, respectively, was lowered onto the left ventricle using a micromanipulator. It was permanently fixed to the heart using a thin layer of clinical grade Vetbond (3M UK plc, Bracknell, UK) on its underside. This sufficiently reduced motion of a small region of the ventricle to allow imaging within its centre whilst the rest of heart continued to beat normally. No BP/HR changes were identified as a result of this process as determined by blood pressure monitoring and photoplethysmograghy (Supplementary material online, *Figure S1*). The central area was kept moist by application of 0.9% saline with the surrounding area covered with saran wrap to prevent loss of moisture. In IRI hearts, application of the stabilizer was initiated five minutes after the LAD artery was unclamped and downstream of the ligation site (area most at risk of infarction). The same region was identified in sham hearts. Intravital imaging was performed using a Nipkow spinning disk confocal head attached to an upright Olympus BX61WI microscope with an Evolve EMCCD camera (Photometrics, USA). Since it took ∼20 to 30 min before the mouse was ready for imaging, the first recording lasting 2 min took place from a pre-selected field of view at 30 min post-reperfusion. Subsequent 2 min recordings were made from the same area every 15 min for 2 h. All data was captured, stored and analysed using Slidebook 6 software (Intelligent Imaging Innovations, USA).

To image endogenous neutrophils and platelets in the same mouse, 20 μL of PE-conjugated rat anti-mouse Gr-1 antibody (RB6-8C5; Thermofisher) and an APC-conjugated rat anti-mouse CD41 antibody (MWReg30, Thermofisher) were both administered 5 min prior to ischaemia. Platelet aggregates/microthrombi were quantitated by placing a mask around CD41 positive areas and integrated fluorescence density, which took into account size and fluorescence intensity, was calculated. Considering the sheer numbers of endogenous platelets, labelling all of them made it difficult to quantitate singular platelet events. Therefore, in separate mice, 1 × 10^8^ CFSE-labelled donor platelets were systemically injected. This results in a labelled fraction in the recipient of ∼5% of all circulating platelets, a value low enough to allow individual platelets to be imaged. To investigate the kinetics of HSPC homing to the heart, 100 μL of 2 × 10^6^ CFSE-labelled HSPCs were injected at 30 min post-reperfusion. This time point enabled stabilizer attachment, mouse transfer to the microscope and HSPC ‘first-pass’ to be imaged. However, to assess potential vasculoprotective effects, HSPCs were injected within 5 min of reperfusion. Neutrophils, platelets and HSPCs were manually counted and considered adherent if they remained static for >30 s. Free flowing cells were identified as those that passed through the coronary microcirculation without making adhesive interactions. To identify areas of perfused blood vessels, 100 μL of FITC-BSA (Sigma, UK) was administered at 2 h post-reperfusion. All intravital data was analysed by an independent blinded observer. At the end of experimentation, mice were culled by cervical dislocation.

### 2.6 Laser speckle contrast imaging of the beating mouse heart

Laser speckle contrast imaging (LSCI) was utilized to quantitate left ventricular myocardial blood flow. Mice were surgically prepared as described earlier but no stabilizer was attached. An LSCI device (moorFLPI-2; Moor Instruments, UK) was positioned above the exposed heart. A demarked area, typically downstream of the LAD ligation site, was identified for collection of flux data during pre-ischaemia, ischaemia and at every 15 min post-reperfusion. A total number of 1000 frames were captured at each time point using the manufacturer supplied image software (mFLPI2Measure V2.0; mFLPIReview V5.0) at a frame rate of 25 Hz and using spatial processing (sliding window, time constant: 0.1 s). *Basic Speckle Analysis software* (SpAn), written in-house (available on request), allowed identification and collation of flux values during diastole for each time point (Supplementary material online, *Figure S2*).

### 2.7 Measurement of serum inflammatory cytokine concentrations

Circulating cytokine concentrations were identified in serum samples from mice following sham surgery, IRI, or IRI+HSPC administration within 5 min of reperfusion. Reperfusion was performed for 1 h. Serum was isolated by centrifugation (2000 rpm, 10 min). Samples were loaded in triplicate onto a Bio-Plex pro mouse cytokine 23-plex assay plate (Bio-Rad Laboratories, CA, USA) and analysed using a Luminex 200 plate reader (Bio-Rad Laboratories, CA, USA) following the manufacturer’s instructions.

### 2.8 Statistical analysis

All statistical analysis was performed using GraphPad Software (GraphPad Software, Inc., USA). Direct comparisons between two groups were performed using a Student’s *t*-test. Comparisons between three or more groups were performed by one-way ANOVA, followed by Sidak *post* *hoc* tests. For time course studies, such as neutrophil recruitment over time, comparisons were made by two-way ANOVA, followed by Sidak *post* *hoc* tests for individual time points. All data is presented as mean ± SEM.

## 3. Results

### 3.1 Neutrophil recruitment and platelet microthrombus formation increases rapidly following myocardial ischaemia–reperfusion injury

**Figure 1:**
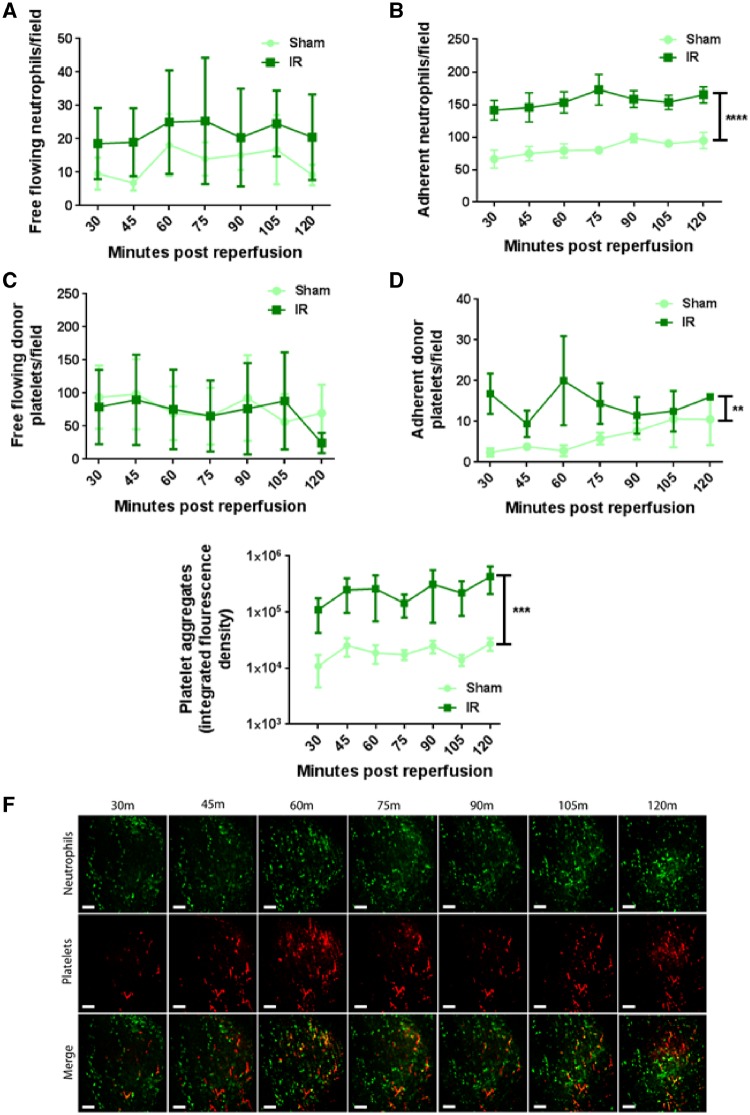
Neutrophil recruitment and microthrombus formation increases rapidly following myocardial IRI. (*A*) Free flowing neutrophil number does not increase following IRI when compared with sham controls (*n* = 5/group). (*B*) Neutrophil adhesion within sham hearts is high but increases following IRI (****P < 0.0001 IR vs. Sham with significant differences at all time points; *n* = 5/group). (*C*) Free flowing donor platelets transmitting through myocardium is not increased following IRI (*n* = 5/group). (*D*) Singular donor platelet adhesion increases across the imaging period following IRI (**P < 0.01 IR vs. Sham; *n* = 5/group). (*E*) Accumulation of endogenous platelets, appearing as platelet aggregates and microthrombi increases following IRI and is quantitated as integrated fluorescence density (***P < 0.001 IR vs. Sham with significant differences at all time points; *n* = 5/group). Two-way ANOVA with Sidak’s multiple comparison test used for all analysis. (*F*) Representative intravital images of the beating mouse heart to show rapid accumulation and gradual increases of both neutrophils and platelets. Neutrophils present primarily as individual cells with platelets found as aggregates or microthrombi. Merged images show aggregates comprised of both cell types (yellow). Green, Neutrophils (PE+anti-Gr-1ab); Red, endogenous platelets (APC+anti-CD41ab). Scale bars represent 100 µm.

The number of neutrophils freely circulating through coronary microcirculation increased at all time points post-reperfusion when compared with sham hearts, but this did not attain statistical significance (*Figure 1A*). Interestingly, numbers of adherent neutrophils within sham hearts was high with ∼60 to 70 cells observed per field of view. However, this more than doubled following IRI and remained significantly (*P* < 0.0001) elevated (*Figure 1B* and *F*). No change in the number of individual, free flowing platelets occurred as a result of injury (*Figure 1C*). Unlike neutrophils, their adhesion within sham hearts was minimal, but significantly (*P* < 0.01) increased following injury (*Figure 1D*). Ischaemia–reperfusion injury also led to the striking and significant (*P* < 0.001) formation of numerous platelet aggregates and microthrombi within coronary capillaries, which was sustained throughout the duration of the experiment (*Figure 1E* and *F*). No such aggregates were identified in sham hearts.

### 3.2 Adherent neutrophils do not compromise blood flow unlike platelet microthrombi

**Figure 2:**
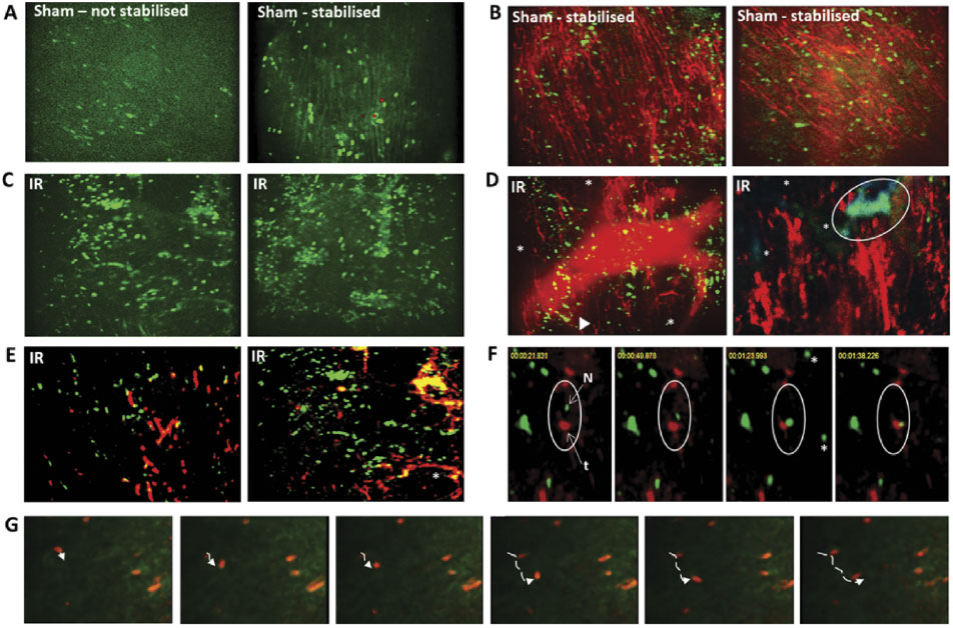
Neutrophils adherent within sham hearts do not prevent blood flow—platelet aggregates main contributors to occlusive events in IRI. (*A*) Blurry and out-of-focus intravital images obtained from ‘non-stabilized’ hearts, but still showing adherent neutrophils in sham myocardial microcirculation. Better quality and easily quantifiable intravital images obtained following stabilization, which confirm neutrophil adhesion in beating sham hearts. (*B*) Adherent neutrophils in sham hearts do not compromise blood flow as widespread FITC-BSA perfused capillaries are visible. Green, neutrophils (PE+anti-Gr-1ab); Red, FITC-BSA. (*C*) IRI increases neutrophil adhesion, most of which are singular, although aggregates also identified. (*D*) Blood flow compromised in IRI hearts as evidenced by not all capillaries being filled with FITC-BSA. This did not always correspond to areas of neutrophil adhesion. Some capillaries ‘finish’ abruptly (arrowhead; out-of-focus large vessel seen in this field of view). Magnified image (right) shows neutrophils often adherent or trapped within platelet microthrombi in injured hearts, as indicated by co-staining, to form large occlusive aggregates. These impact detrimentally on blood flow as indicated by inability of FITC-BSA (red) to perfuse into capillaries (*) downstream of microthrombus (blue/green—circle) comprised of platelets (blue) and neutrophils (green). (*E*) Neutrophil adhesion, platelet aggregation and microthrombus formation following IRI. Microthrombi occupy and follow the contours of a significant length of the capillary (*). Co-localization (yellow) indicates aggregates are often, but not always, comprised of both neutrophils and platelets. Green, neutrophils (PE+anti-Gr-1ab); Red, endogenous platelets (APC+anti-CD41ab). (*F*) Time-lapse images from an IRI beating heart video. From left to right, within the white circles, a single circulating neutrophil (green, N) is shown unable to move down the capillary as it is occluded by a downstream stationary platelet thrombus (red, t). *Circulating neutrophils seen in one frame but not the next. (*G*) Time lapse images from a sham beating heart showing a ‘patrolling’ neutrophil. Dotted lines—track movement of ‘patrolling’ neutrophil. Scale bars represent 100 μm.

**Figure 3:**
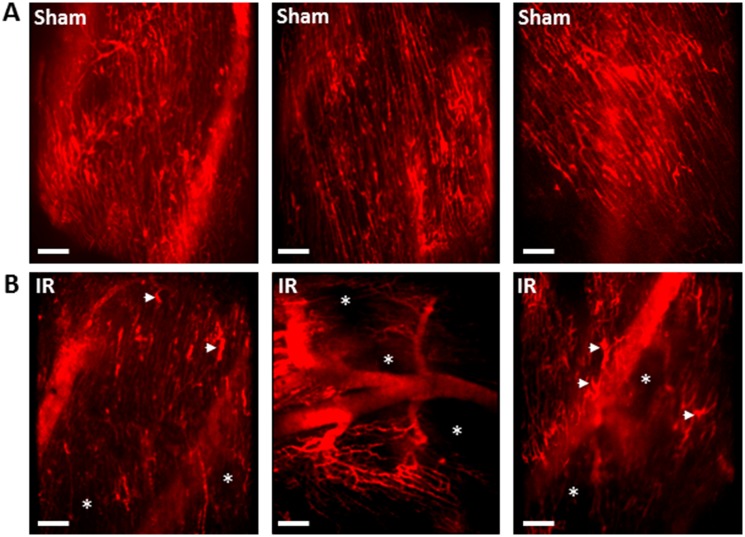
Myocardial IRI is associated with significantly impaired capillary perfusion and a disturbed microvascular organization. FITC-BSA was administered 2 h post-reperfusion and representative intravital images are shown. (*A*) In sham mice, an extensive network of FITC-BSA perfused capillaries can be observed. Most capillaries parallel the arrangement of the muscle fibres with cross connections that run obliquely to fibres along their length. Although the stabilizer is attached to avoid visible large coronary arteries, well perfused medium-sized vessels can be seen in some fields of view (out of focus in some of the images). (*B*) IRI is associated with multiple areas in which FITC-BSA does not perfuse, resulting in visualization of patchy areas devoid of any vasculature (*). In some fields of view, at least half the imaged area appears non-perfused. The organized parallel arrangement of capillaries is lost with microvasculature appearing ‘disorganized’. Accumulation of FITC-BSA, identified as wider areas of intense fluorescence, occasionally seen along the length of some capillaries (arrowheads). Interestingly, medium sized vessels are still perfused and thus readily visible. Scale bars represent 100 µm.

Since significant adherent neutrophils were observed in sham hearts, it was possible that the process of placing the stabilizer induced a local inflammatory response. Therefore, sham experiments were conducted mice in which the beating heart was imaged in the absence of the stabilizer. Although images were difficult to obtain, blurry and relatively out-of-focus, adherent neutrophils were still present in similar numbers to those seen in stabilized hearts (*Figure 2A*). They did not compromise blood flow as evidenced by FITC-BSA perfused capillaries being visible in the whole field of view (*Figures 2B* and *3A*). Ischaemia–reperfusion injury induced a marked and significant increase in individual neutrophil adhesion, although neutrophil clusters were also identified (*Figure 2C*). Blood flow was compromised in IRI hearts as evidenced by a failure of some capillaries to load with FITC-BSA (*Figures 2D* and *3B*). Interestingly, these areas did not correspond with neutrophil adhesion, but rather predominantly matched areas in which striking adherent platelet aggregates and microthrombi were identified. These were occlusive and often identified upstream of areas in which no FITC-BSA was observed (*Figure 2D* and *E*). Microthrombi were not generally observed in the larger vessels but formed primarily within coronary capillaries often occupying significant lengths of vessel. Numerous smaller and more rounded platelet aggregates were also found dispersed within the field of view. Aggregates were often, but not always, comprised of both neutrophils and platelets. These aggregates appeared to occasionally ‘block’ the circulation of trafficking neutrophils (*Figure 2F*). Although adherent neutrophils appeared at first glance to be completely stationary, detailed analysis of videos demonstrated that some neutrophils, in both sham and IRI hearts, were actually moving short distances or ‘patrolling’ the length of the capillary (*Figure 2G*).

### 3.3 Myocardial ischaemia–reperfusion injury is associated with impaired capillary perfusion

An extensive network of FITC-BSA perfused capillaries was observed in sham mice, paralleling the arrangement of muscle fibres, with cross connections along their length. Focusing up and down on the field of view showed no areas devoid of perfused capillaries. Well perfused medium-sized vessels were also visible in some fields of view (*Figure 3A*). In contrast, IRI was associated with multiple areas in which FITC-BSA did not perfuse. This resulted in patchy areas that appeared devoid of any microvasculature, indicating poor FCD. Indeed, in some fields of view, up to half the imaged area appeared non-perfused. Furthermore, the structured parallel arrangement of capillaries was lost with the microvasculature appearing more disorganized. Accumulation of FITC-BSA, identified as slightly wider vascular areas of intense fluorescence, was also seen along the length of some capillaries. Interestingly, medium sized vessels were still readily visible and well perfused in injured hearts (*Figure 3B*).

### 3.4 Despite poor haematopoietic stem/progenitor cell retention within injured hearts, significant vasculoprotective effects are identified

**Figure 4:**
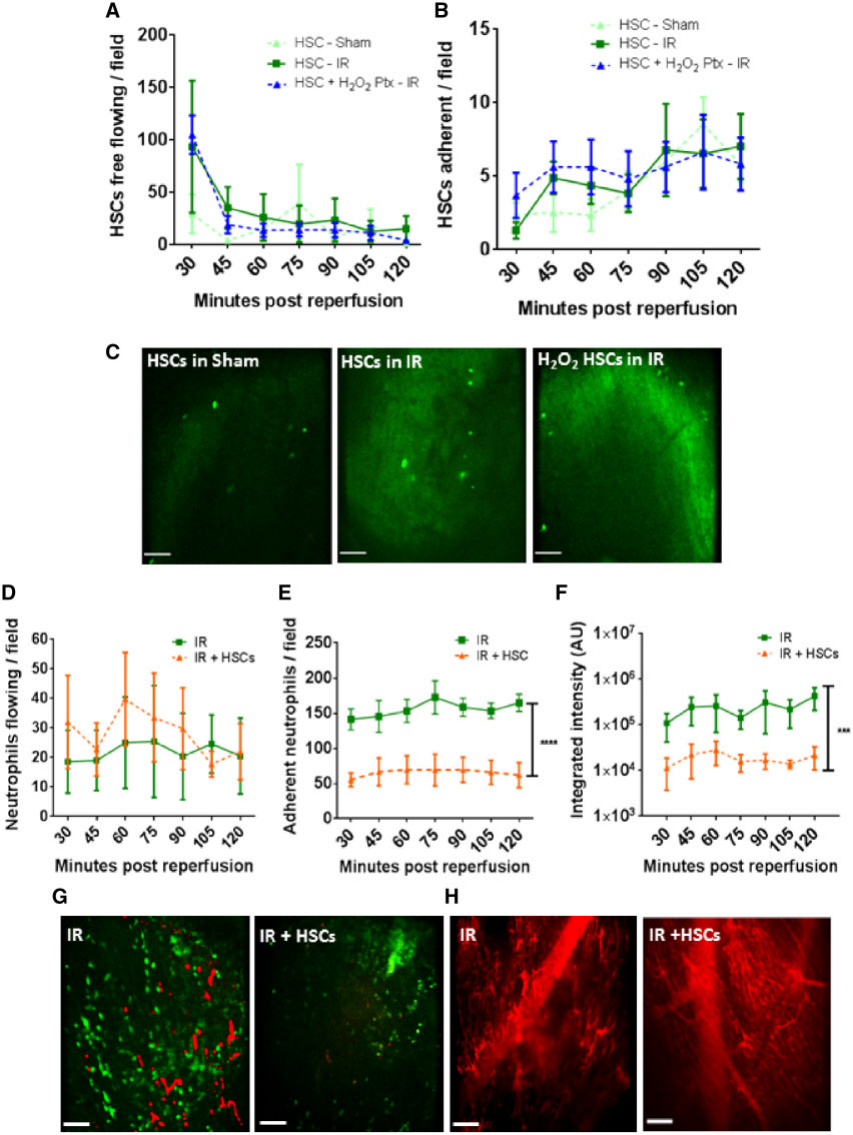
Despite poor HSPC retention within IRI hearts, vasculoprotective effects are identified. (*A*) Twenty to 30 free flowing HSPCs observed circulating through myocardial microvessels at all time points in sham mice. However, in IRI hearts, an almost four-fold increase is noted at 30 min post-reperfusion. Pre-treatment of HSPCs with H_2_O_2_ does not enhance their homing to injured heart. (*B*) Adhesion of naïve or pre-treated HSPCs is not increased as a result of injury but gradually rises in all groups. (*C*) Representative intravital images show a similar retention of intra-arterially injected exogenous CFSE-labelled HSPCs in all groups. (*D*) HSPCs do not affect the free flow of neutrophils through the injured heart but do decrease (*E*) neutrophil adhesion (****P < 0.0001 vs. IR with significant differences at all time points; *n* ≥ 4). (*F*) Presence of platelet aggregates and microthrombi is also significantly reduced (***P < 0.001 vs. IR with significant differences at all time points; *n* ≥ 4). Two-way ANOVA with Sidak’s multiple comparison test used for all analysis. (*G*) Representative intravital images of injured heart showing a reduction in endogenous neutrophils and platelet presence when compared with non-treated injured hearts. Green, neutrophil (PE+anti-Gr-1ab); Red, endogenous platelets (APC+anti-CD41ab). (*H*) Improvement in capillary perfusion subsequent to cellular therapy as determined using FITC-BSA. Scale bars represent 100 µm.

Approximately 20–30 free flowing HSPCs were identified at all time points in sham mice. However, in IRI hearts, this number increased almost four-fold at 30 min post-reperfusion (the first time point after infusion). At all other time points, although circulating HSPC numbers remained elevated, the effect was not as remarkable. H_2_O_2_ pre-treatment did not enhance HSPC homing to the injured heart (*Figure 4A*). Adhesion was low and did not differ between IRI and sham hearts nor with pre-treatment (*Figure 4B* and *C*). HSPC infusion did not affect circulating neutrophil numbers but did remarkably and significantly decrease neutrophil (*P* < 0.0001) and platelet (*P* < 0.001) adhesive events in IRI hearts (*Figure 4D*–*G*). This resulted in clear improvements in capillary FITC-BSA perfusion (*Figure 4H*). Since H_2_O_2_ did not influence HSPC homing, the vasculoprotective effects of pre-treated cells was not tested.

### 3.5 Myocardial ischaemia–reperfusion injury induced a sustained ventricular hyperaemic response

**Figure 5:**
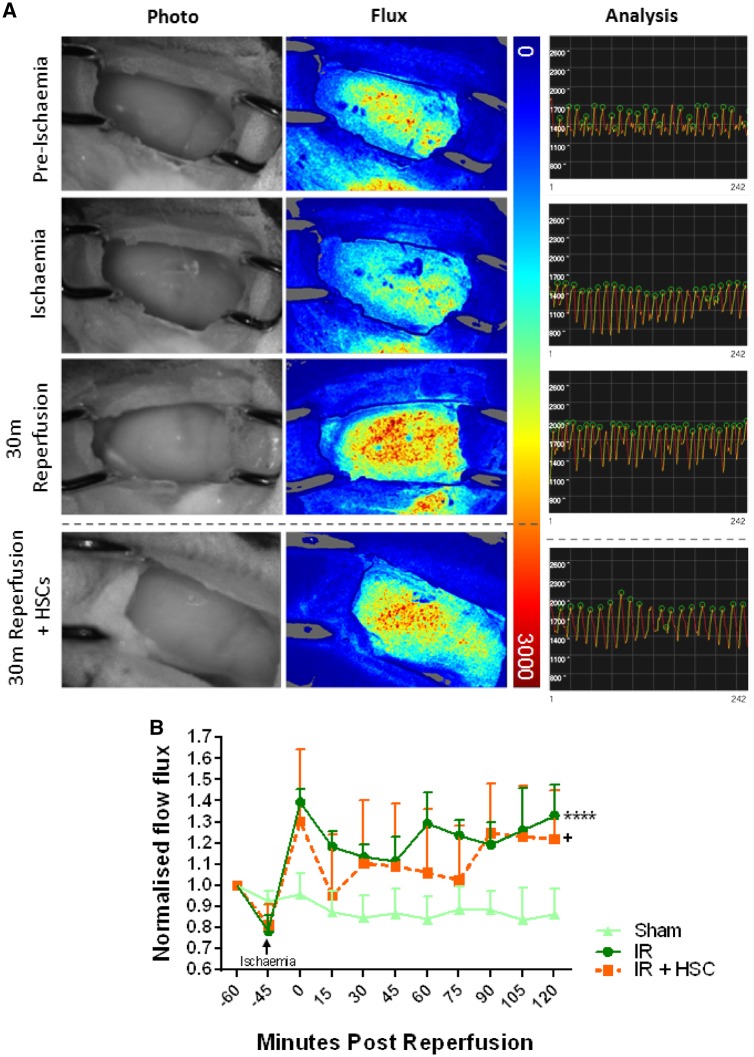
Laser speckle contrast imaging (LSCI) reveals a sustained reactive myocardial hyperaemia in response to IRI. (*A*) Photos of the beating mouse heart are shown alongside the corresponding full field LSCI flux heat map. All flux images have been normalized on the same palette settings. Pre-ischaemia hearts are well perfused as indicated by a more yellow/red heat map. As expected, flow is reduced in the ischaemic heart resulting in heat maps that are primarily blue. Reperfusion induces a rapid and striking hyperaemic response as indicated by mostly red heat maps. Analysis graphs from SpAN rendered data show diastolic and systolic events captured as high points (green circles) and low points, respectively. In these representative analysis graphs, arbitrary flux values decrease from ∼1700 to ∼1400 as a result of ischaemia and increase to ∼2000 after reperfusion. (*B*) This raw data has been presented as mean normalized flow flux. In sham mice, ventricular blood flux does not change throughout the course of imaging. Ischaemia decreases this value. Reperfusion induces a rapid and sustained reactive hyperaemic response. The presence of HSPCs in IRI mice produces flux recordings statistically different to sham hearts but no different to IRI hearts not receiving HSPCs. ****P < 0.0001, ^+^*P* < 0.05; ANOVA with Sidak’s multiple comparison test; *n* = 3/group.

Left ventricular diastolic and systolic events were easily detected on flux analysis recordings as high points and low points, respectively. We focused on diastolic events for comparative purposes (*Figure 5A*). In sham mice, blood flow did not change throughout the course of imaging. As expected, ischaemia reduced flow with arbitrary flux values decreasing from ∼1700 to ∼1400. Reperfusion was associated with a significant (*P* < 0.001) and sustained reactive hyperaemic response with values increasing to ∼2000 (*Figure 5B*). The presence of HSPCs in IRI hearts produced blood flow flux recordings that were statistically (*P* < 0.05) different to sham hearts but did not differ from IRI hearts not receiving HSPCs.

### 3.6 Myocardial endothelial cells are more susceptible to oxidative stress than cardiomyocytes following acute ischaemia–reperfusion injury but this injury can be reduced by haematopoietic stem/progenitor cells

**Figure 6:**
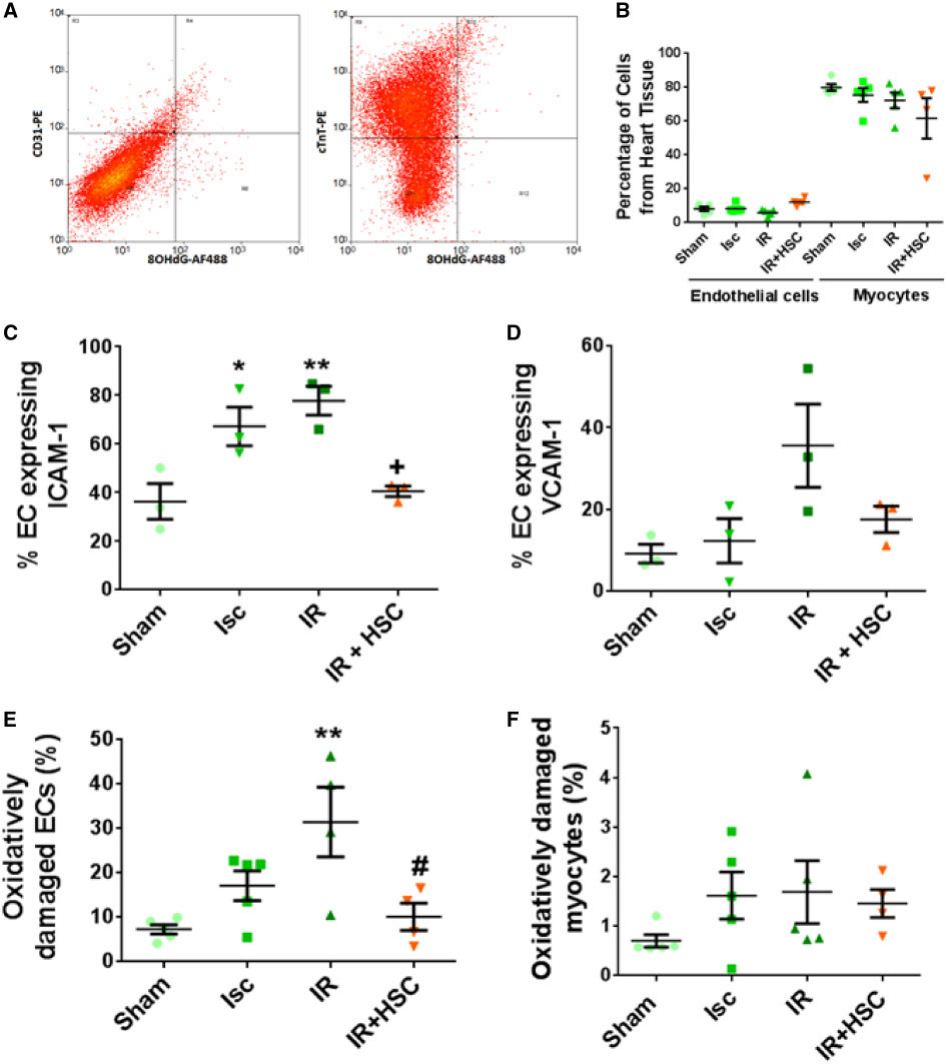
Myocardial endothelial cells are more susceptible than myocytes to oxidative stress following acute IRI but this injury can be reduced by HSPCs. (*A*) Flow cytometric gating strategy used for identifying oxidatively damaged (8-OHdG^+^) ECs (CD31^+^; left) and myocytes (cTnT^+^; right) in digested whole hearts. (*B*) Percentage of each cell type obtained from a total of 30 000 events. A greater yield of myocytes is obtained (*n* = 5; IR+HSPC groups *n* = 4). (*C*) Endothelial ICAM-1 expression increases during ischaemia and further during reperfusion. This is reduced by HSPCs injected at the onset of reperfusion (*P < 0.05, **P < 0.01 vs. sham; ^+^*P* < 0.05 vs. IR; Sidak; *n* = 3). (*D*) Endothelial VCAM-1 expression does not increase during ischaemia but non-significantly increases during reperfusion. This is also reduced following HSPC administration (Sidak; *n* = 3). (*E*) IRI, but not ischaemia alone, results in a significant endothelial oxidative damage which is reduced with HSPCs (**P < 0.01 vs. sham; ^#^*P* < 0.05 vs. IR; Sidak; sham and ischaemia groups *n* = 5; IR and IR+HSPC groups *n* = 4). (*F*) Oxidative damage in myocytes also increases but not to levels seen in endothelium (Sidak; *n* = 5; IR+HSPC group *n* = 4).

Digested whole heart cells were examined using flow cytometry and CD31^+^ ECs and CTnT^+^ myocytes identified (*Figure 6A*). The percentage of myocytes recovered from the sampled population of cardiac cells was consistently higher (∼80%) than the yield of ECs (∼10%) across all groups of mice (*Figure 6B*). Compared with sham controls, ICAM-1 expression on ECs was significantly increased during ischaemia (*P* < 0.05) and reperfusion (*P* < 0.01). This increase was reduced in mice receiving HSPCs when compared with IRI mice not receiving cellular therapy (*Figure 6C*). Interestingly, VCAM-1 and oxidative stress (*P* < 0.01) only increased during reperfusion with both returning to baseline values in mice receiving HSPCs (*Figure 6D* and *E*). Although there was a trend for myocytes to also undergo oxidative damage following injury, this did not reach statistical significance. Furthermore, only ∼1 to 2% of myocytes presented with oxidative damage compared with ∼30% of ECs (*Figure 6F*).

### 3.7 Microvascular endothelial oxidative stress can be reduced in the presence of haematopoietic stem/progenitor cells *in vitro*

**Figure 7:**
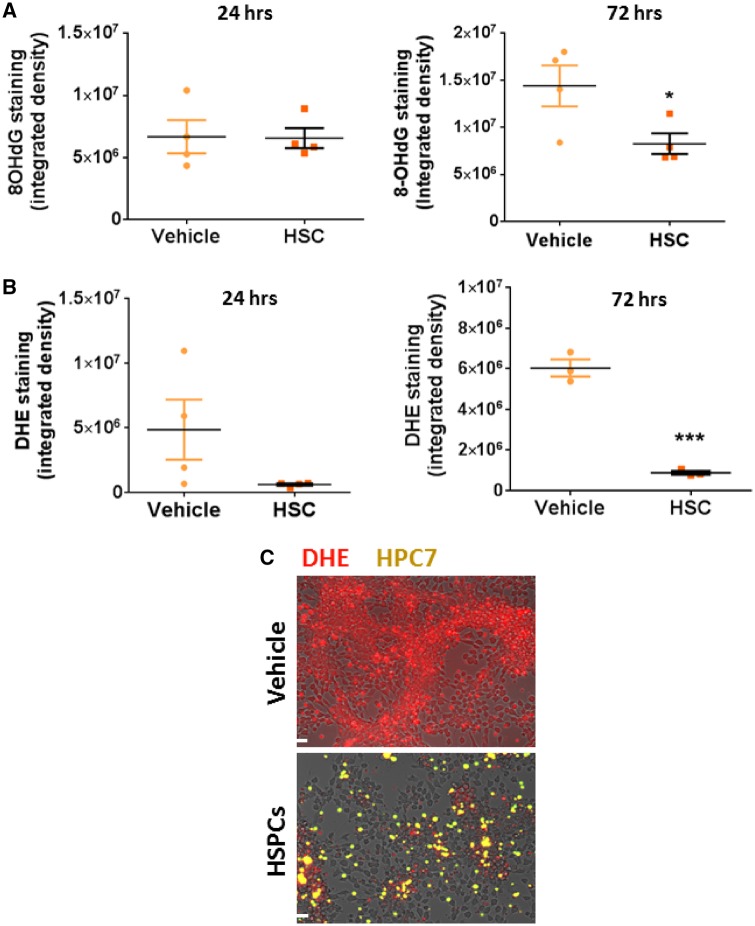
Microvascular endothelial oxidative stress can be reduced in VCECs in the presence of HSPCs *in vitro*. (*A*) Oxidative damage incurred by H_2_O_2_ treated vena cava endothelium is reduced following incubation with HSPCs for 72 h (*P < 0.05; *n* = 4; *t*-test). (*B* and *C*) H_2_O_2_ driven ROS generation, measured by DHE staining, is reduced by HSPCs at 72 h (***P < 0.001; *n* = 3; *t*-test). Red, DHE stain; Yellow, CFSE-labelled HSPCs. Scale bars represent 100 μm.

The ability of HSPCs to decrease oxidative stress, specifically in cardiovascular ECs *in vitro*, was tested in a co-culture system using VCECs. Oxidative damage resulting from H_2_O_2_ treatment, determined by 8-OHdG staining, was not reduced following incubation with HSPCs for 24 h but was significantly (*P* < 0.05) reduced by 72 h (*Figure 7A*). H_2_O_2_ driven ROS generation, measured by DHE staining, was also reduced at 72 h (*P* < 0.001) following incubation with HSPCs (*Figure 7B* and *C*).

### 3.8 Haematopoietic stem/progenitor cells significantly decreased serum levels of multiple pro-inflammatory cytokines but increased levels of interleukin-10

Ischaemia–reperfusion injury induced a significant (*P* < 0.05) increase in the circulating serum levels of 10 out of 23 inflammatory cytokines tested (*Table 1*). Greater than six-fold increases were observed in interleukin (IL)-6, IL-12, G-CSF, KC, MCP-1, and MIP-1β levels. Haematopoietic stem/progenitor cells reduced the serum concentration of nine of these factors as well as some cytokines whose increase post-IRI was not significantly increased. Interestingly, HSPCs significantly (*P* < 0.01) increased the serum levels of IL-10.

**Table 1:** HSPCs reduce serum concentration of multiple pro-inflammatory factors but increase serum levels of IL-10, a known anti-inflammatory cytokine

Cytokines	Sham (pg/mL)	IR injury (pg/mL)	IR injury + HSCs (pg/mL)
Interleukin-1α	14.07 ± 2.90	18.62 ± 3.95	10.63 ± 0.67*
Interleukin-1β	37.18 ± 5.06	42.84 ± 4.37	24.94 ± 2.40**
Interleukin-2	14.92 ± 3.00	11.65 ± 0.86	8.17 ± 0.48**
Interleukin-3	17.13 ± 2.45	16.46 ± 1.67	10.93 ± 0.93**
Interleukin-4	13.61 ± 3.48	10.27 ± 2.77	6.76 ± 4.29
Interleukin-5	32.63 ± 11.95	31.56 ± 5.69	17.78 ± 2.91*
Interleukin-6	316.138 ± 118.21	1993.53 ± 841.45*	425.07 ± 78.19*
Interleukin-9	51.29 ± 7.54	45.76 ± 4.83	34.22 ± 2.64*
Interleukin-10	143.57 ± 9.06	222.70 ± 45.84*	452.48 ± 41.06***
Interleukin-12(p40)	2253.75 ± 350.50	14 588.86 ± 5889.30*	2424.66 ± 179.39*
Interleukin-12(p70)	501.84 ± 85.43	555.36 ± 57.31	350.24 ± 38.07**
Interleukin-13	278.182 ± 51.10	337.87 ± 38.67	159.26 ±25.87***
Interleukin-17	18.70 ± 2.68	14.50 ± 1.22	11.36 ± 1.06*
Eotaxin	992.67 ± 113.83	1607.58 ± 50.43*	1020.15 ± 90.44***
G-CSF	395.23 ± 97.79	2720.72 ± 1272.80*	200.29 ± 36.04*
GM-CSF	54.52 ± 7.25	61.95 ± 6.50	41.09 ± 4.75**
IFNγ	46.44 ± 7.28	42.25 ± 4.50	30.55 ± 2.20*
KC (IL-8)	326.15 ± 93.22	1769.48 ± 783.47*	227.52 ± 28.93*
MCP-1	638.92 ± 121.67	5016.62 ± 2117.35*	704.97 ± 93.02*
MIP-1α	21.84 ± 8.95	48.51 ± 21.69	37.30 ± 10.35
MIP-1β	169.46 ± 56.60	1545.83 ± 736.81*	250.28 ± 58.80*
RANTES	108.06 ± 15.03	248.96 ± 79.09*	85.75 ± 10.29*
TNFα	191.95 ± 29.16	369.49 ± 95.21*	154.62 ± 19.28*

IRI induced a significant increase in the circulating serum levels of 10 out of 23 inflammatory cytokines tested using a Luminex cytokine array kit. Greater than six-fold increases were observed in IL-6, IL-12, G-CSF, KC, MCP-1, and MIP-1β levels. HSPCs reduced the serum concentration of nine of these factors as well as some cytokines whose increase post-IRI was not significantly increased. HSPC administration significantly (*P* < 0.01) increased the serum levels of interleukin-10.

* *P* < 0.05,

** *P* < 0.01,

*** *P* < 0.001;

Sham vs. IR injury or IR injury with HSPCs. One-tailed Student’s *t*-test, *n* > 9.

## 4. Discussion

Adequate microcirculatory perfusion, and not just opening of an occluded epicardial artery, is critical in order to salvage myocardial tissue post-MI. However, the pathophysiological changes in coronary microvascular perfusion taking place following an ischaemic insult have not previously been imaged directly. Here, we present a novel approach to image multiple coronary microcirculatory perturbations intravitally in a stabilized beating mouse heart with assessment of overall blood flow using laser speckle imaging. A sustained ventricular hyperaemic response was noted throughout reperfusion. On its own, this could have been interpreted as blood flow being adequately re-established to repay the debt acquired during ischaemia. However, it was clear from intravital studies that this was poorly transmitted to coronary capillaries and thus did not correspond to adequate perfusion at a microvascular level.

Neutrophil adhesion increased rapidly in injured hearts, doubling by 30 min post-reperfusion. Neutrophil capture is generally confined to post-capillary venules due to their high and preferential expression of endothelial adhesion molecules.^22^ In the heart, we noted neutrophil retention mainly within capillaries, a region of the vasculature not usually associated with cellular recruitment. VCAM-1 expression was also increased in IRI vasculature. However, unlike in previously published work,^23^ this was observed on capillaries as well as larger vessels. This suggests active capture mechanisms may also be involved in mediating capillary-neutrophil interactions in the heart. Neutrophil accumulation has long been recognized histologically as a significant component of myocardial IRI. However, the impact of their sequestration on coronary perfusion has not been possible to determine from static sections. Intravitally, we show that obstructions in microvessel blood flow do not generally occur in areas with individual adherent neutrophils. This was evidenced by the fact that FCD did not decrease in the whole field of view in parallel with the diffuse neutrophil adhesion. Indeed, only distinct patchy areas lacking FITC-BSA perfusion were observed.

Interestingly, a significant population of adherent neutrophils were observed in sham hearts *in vivo*. This was unusual based on our extensive intravital experience of imaging other solid organs.^15–19^ Adhesion many have been stimulated by attaching the stabilizer but this is unlikely as they this population was present even in non-stabilized hearts. We speculate that some trafficking neutrophils may have become trapped as blood vessels were compressed during systole. Extravascular compression during isovolumetric contraction markedly reduces coronary flow.^24–26^ Indeed, LSCI clearly identified decreased myocardial blood flow during systole. As far as we are aware, this is the first application of LSCI to the beating mouse ventricle and demonstration of real-time phasic changes associated with the cardiac cycle. Interestingly, adherent neutrophils did not obstruct blood flow as evidenced by the ease and ability of FITC-BSA to permeate through the sham heart microvasculature.

Numbers of free-flowing neutrophils circulating through the injured heart also doubled throughout reperfusion. This is an interesting observation, particularly in light of the fact that the density of FITC-BSA perfused capillaries decreased. Higher numbers of circulating neutrophils passing through less microvasculature can be explained by a possible increase in the blood flow to the injured heart. Indeed, this is supported by the LSCI data. Reactive hyperaemic responses post-occlusion benefit the oxygen-deprived myocardium. However, these responses also inadvertently introduce more inflammatory cells into an environment primed for their adhesion.

We also occasionally observed neutrophils, in both sham and injured hearts, which appeared to slowly ‘patrol’ short lengths of the capillary. This patrolling behaviour is novel in the heart and the physiological relevance is unclear in sham mice, but following IRI it may possibly reflect attempts to identify emigration sites. Recent intravital imaging provides evidence that neutrophils also constitutively patrol glomerular capillaries and following injury exhibit a greater dwell time and increased ROS generation.^27^ ‘Crawling’ neutrophils, seeking to provide the first line of defense against infection, have also been identified in pulmonary capillaries.^28^ Collectively, these studies provide insights into a new neutrophil behaviour that does not appear to be site-specific.

Although individual platelet adhesion was enhanced, it was the formation and sustained elevated presence of platelet aggregates that was a striking feature in reperfused hearts. Numerous small, rounded aggregates were seen as well as larger, elongated microthrombi occupying significant lengths of the capillaries. The major consequence of these was the detrimental effect on capillary perfusion in their immediate vicinity. This was demonstrated by the lack of FITC-BSA presence downstream of microthrombi. Hence multiple non-perfused areas were identified interspersed with perfused microvasculature. Although it is often suggested that platelet microaggregates impede microvascular flow following MI,^29^^,^^30^ our studies provide the first direct *in vivo* evidence of their occlusive nature in an IRI beating heart.

A marked but transient increase in blood delivery to the heart following ischaemia, lasting only a few minutes, has been demonstrated both experimentally and clinically.^31^^,^^32^ Indeed in humans, coronary blood flow can increase up to five times basal flow to meet increased demand following release of a variety of vasodilatory factors.^31^ Interestingly, in the current study a more sustained reactive hyperaemic response was identified. This discrepancy may possibly be explained by technical differences. Previously, blood flow responses were measured from one of the major coronary arteries using an inserted Doppler flow guide wire. However, in the current study, the full-field optical nature of LSCI was able to image a larger ventricular area, thus averaging macro- and microvascular flow changes, over longer periods of time.

Although individual neutrophils did not appear to hinder flow, their increased presence is detrimental. Since activated neutrophils generate ROS, their rapid and high capillary presence increases the susceptibility of these delicate structures to early and significant oxidative damage. Indeed, flow cytometry data demonstrated coronary endothelium, rather than myocytes, was the primary and initial target of oxidative injury. Furthermore, a high degree of correlation between capillary CD31 and 8-OHdG immunostaining was noted histologically. Others have also shown this differential susceptibility to injury with endothelial and cardiomyocyte apoptotic death occurring at 5 and 60 min, respectively in isolated perfused hearts.^33^^,^^34^ Collectively, this data highlights coronary microcirculation as an early and principal therapeutic target to prevent ensuing muscle damage.

Our model was therefore used for the first time to image stem cell trafficking *in vivo* at a cellular level and, importantly, ascertain whether vasculoprotection was a critical therapeutic mechanism. We focused on cellular therapy as there has been intense interest in their use for cardiovascular diseases. Even through the field has progressed rapidly to clinical trials, it is agreed that more basic research is required to understand their mechanisms of action and thus improve on their modest clinical success. Although clinical studies have primarily used total un-fractionated BM mononuclear cells [comprising HSPCs, mesenchymal stem cells (MSCs), lymphocytes and monocytes], we focused on a purified population of HSPCs. A four-fold increase in circulating HSPCs was identified in IRI hearts, which may be related to the hyperaemic response. However, 25–30 circulating cells were consistently observed throughout reperfusion. This was unusual as we have shown in other organs that HSPCs were only observed on the ‘first pass’ immediately after infusion and not thereafter.^16–18^^,^^20^ Surprisingly, these homing events did not result in any dramatic local HSPC retention, which remained low and similar to sham hearts. Furthermore, pre-treatment strategies did not modify their adhesion. Poor retention is a well described phenomenon. For example, Hofmann *et al*.^35^ demonstrated <2.6% retention of unselected BM-derived cells in ST-segment elevation MI patients when delivered 5–10 days after stenting. Pulmonary entrapment is a common feature of cellular therapy^36^^,^^37^ which we also noted in the current study.

This observation was potentially troublesome, as the efficacy of cellular therapy has been thought to be dependent on sufficient retention within injury sites.^38^ Indeed, a recent study demonstrated that developing platelet-based strategies to improve the targeted delivery of peripheral blood mononuclear cells to the IRI heart led to reduced infiltration of inflammatory cells, reduced fibrosis and enhanced capillary density after IRI.^39^ However, we showed that despite limited local retention, HSPCs afforded rapid and remarkable vasculoprotection in the injured heart, limiting both neutrophil and platelet adhesion and thus improving FCD. Bone marrow-derived stem and progenitor cells can secrete a rich and potent combination of growth factors and anti-inflammatory cytokines that act in a paracrine manner on neighbouring cells.^40^^,^^41^ Even without retention in the heart, these factors may be released systemically from remote sites and confer vasculoprotection in the heart.^36^ Indeed, remote transplantation of MSCs into the interscapular region protected the IRI heart.^42^ Since HSPCs continuously circulated through the heart, it is also possible they became activated to release paracrine factors locally as they trafficked through the injured coronary microcirculation. Haematopoietic stem/progenitor cells inhibited both a number of pro-inflammatory cytokines as well as ICAM-1/VCAM-1 up-regulation, which could mechanistically explain the decreased neutrophil infiltration. Interestingly, we show that HSPCs also induced a significant increase in circulating IL-10 levels in IRI mice. IL-10 is a naturally occurring, potent anti-inflammatory cytokine which can suppress secretion of various pro-inflammatory cytokines both *in vitro* and *in vivo* via inhibition of NF-κβ.^43^ A number of immunohistochemical studies have demonstrated that exogenous IL-10 can suppress myocardial inflammation as evidenced by decreased neutrophil infiltration in similar injury models.^44^^,^^45^ Indeed, the beneficial effects of remote ischaemic pre-conditioning in the heart have also been demonstrated to be mediated via increases in IL-10.^46^ Furthermore, genetically engineering mesenchymal stem cells (MSC) to overexpress IL-10 has been shown to better reduce infarct size and cardiac impairment compared with individual treatments of MSC or IL-10 administration.^47^ It is therefore likely that increased serum levels of IL-10 in mice receiving HSPCs mechanistically explains the vasculoprotective effects observed in the current study.

The impact on platelet activity is somewhat harder to explain. It is possible that platelet aggregation occurred subsequent to endothelial damage and denudation instigated by neutrophils. However, since both thrombotic and inflammatory events had already occurred by the earliest imaging time point, it is not clear which cellular event occurred first. Alternatively, HSPCs may possess direct anti-thrombotic potency. Indeed, this was recently described for MSCs in the lungs and was linked to modification of urokinase-type plasminogen activator, a protein important in early thrombi resolution.^48^^,^^49^ Whether HSPCs modify this pathway is not known and would require further examination. The only event not influenced by HSPCs was hyperaemia, which is perhaps not surprising, as this is a reactive response to ischaemia which was induced in all experimental groups including mice receiving cellular therapy.

Spinning disk confocal allowed for high resolution and real-time capture of dynamic images of the beating heart that would not have been possible with conventional single laser raster scanning microscopy. However, the imaging is relatively ‘superficial’ with an imaging depth of ∼25 to 35μm beneath the heart surface. Theoretically, confocal imaging could have delivered signals from more depth. However, our low exposure times, required for video-rate imaging of coronary microvessels, limited imaging depth. Greater depth could be achieved with multiphoton microscopy. However, our initial attempts using multiphoton IVM (Olympus Fluoview) have been challenging due to residual movement that remains even after stabilization (unpublished data) and additional methodological enhancements are required such as respiratory/cardiac gating. Even so, we find imaging depth in the heart to be modestly increased to ∼70 to 80μm. To appreciate the global effects of myocardial IRI on the coronary microcirculation throughout the muscle wall, multiphoton imaging on tissue sections spanning the full thickness of the ventricular wall is required.

To summarize, intravital imaging of the beating mouse heart, combined with laser speckle microscopy, provides a wealth of dynamic information on the coronary microcirculation that is not possible to obtain with conventional immunohistological approaches. We show for the first time that although IRI is associated with an overall hyperaemic response in the injured ventricle, this may be deceptive as at a microvascular level, increased flow heterogeneity exists due to occluded coronary capillaries. Hence there exists a great mismatch between a ‘global hyperaemic response’ during reperfusion, and microcirculatory heterogeneity. To the best of our knowledge, this is the first detailed *in vivo* characterization of multiple microcirculatory perturbations taking place in the IRI beating heart. Although Li *et al*.^15^ have also intravitally imaged a beating mouse heart, they imaged a donor heart transplanted in the neck of the recipient, with a focus on neutrophil kinetics. Crucially, our research is conducted on the native heart *in situ* which has greater clinical relevance as an experimental model for MI. We further show that local stem cell presence is not a pre-requisite to preventing myocardial thromboinflammatory events. Since acellular delivery of stem cell-derived paracrine factors may be sufficient to activate repair mechanisms, this should be tested in our model and is indeed the basis of future studies. It is anticipated that this model will have a number of future applications. For example, microvascular dysfunction is a key feature of cardiovascular disease in females, with both cardiac syndrome X and MINOCA being more common in women. Applying our beating heart intravital imaging method to female mice would be a worthwhile pursuit to assess sex related differences in the microcirculatory responses to IR injury.

## Supplementary Material

cvz118_Supplementary_Data

## Notes

**Time for primary review: 25 days**
