# Supplementary material for: Imaging the injured beating heart intravitally and the vasculoprotection afforded by haematopoietic stem cells
Source: Cardiovasc Res. 2019 May 7;115(13):1918–32. doi: 10.1093/cvr/cvz118 (PMC6803816; doi:10.1093/cvr/cvz118)
Supplement: cvz118_Supplementary_Data [file cvz118_supplementary_data.zip › cvz118-Suppl_data/Supplementary_Data.pdf]

Imaging the injured beating heart intravitally and the vasculoprotection afforded by haematopoietic stem cells

Dean P.J. Kavanagh* (PhD), Adam Lokman* (MBBS), Georgiana Neag (MRes), Abigail Colley (MRes), Neena Kalia (PhD)

*** Joint First Authors**

SUPPLEMENTARY FIGURE LEGENDS, SUPPLEMENTARY VIDEO LEGENDS and SUPPLEMENTARY FIGURES

Supplementary Figure 1. Fluorescent intravital microscopy imaging of the mouse beating heart microcirculation is successfully achieved through stabilisation of a small region of the left ventricle. (A-C) An in-house designed 3D printed stabiliser is vet-bonded to the beating left ventricle of the anaesthetised mouse allowing Spinning Nipkow confocal based intravital imaging in its centre. In LAD ligated and reperfused hearts, the stabiliser is attached downstream of the ligated site. The stabiliser carefully approaches the exposed heart with the aid of a precision micromanipulator. The internal and external diameter of the stabiliser is 2.25mm and 4mm respectively and so only a small surface of the heart has its motion reduced or stabilised sufficiently enough to permit imaging. The central region is kept moist by topical application of warmed saline. During distole, the tissue is still. However, during contraction, motion is significantly reduced but not enitrely inhibited in the centre of the ‘stabilised’ region. Nipkow-spinning disk confocal provides up to 10,000 parallel and simultaneous excitation laser beams which allows for a high signal-to-noise ratio such that absolute stillness is not critical. Images of sufficient quality can be obtained provided that the time dimension of the imaging window is set sufficiently high to allow for the capture of still events, but short enough to prevent blurring (we use 50ms, a frame rate of 20Hz). **(D)** No BP/HR changes were detected using this approach as determined by photoplethymosography in both sham and IR injured mice. The graph presented shows blood pressure which remains constant after the stabiliser is attached. **(E)** An Olympus BX61-WI upright intravital microscope with spinning Nipkow confocal capabilities is used to image the heart in the centre of the stabiliser with images captured using a 10x dry objective and an EMCCD camera.

Supplementary Figure 2. Full-field laser speckle contrast imaging (LSCI) and subsequent analysis of the beating mouse heart blood flow following myocardial IR injury. (A) The non-static physical nature of the heart, and the dynamics of microvascular blood flow during systole and diastole, present challenges for interpretation of laser speckle images of ventricular muscle blood flow. However, we show that diastole and systole present characteristically during LSCI recordings as peaks/high points and troughs/low points respectively. These can be clearly seen on the analysis graphs. Left panel: Photo; Middle panel: Flux image; Right panel: Flux graph for the area demarked on the flux image. The red line passes through a trough in the upper analysis image and a peak in the lower analysis image. When correlating to the flux image, it is clear that these represent a time point when the heart is poorly and highly perfused respectively. **(B)** The nature of diastole would suggest that the high peaks are obtained during this point of the cardiac cycle. The chamber blood volume is high due to ventricular filling and myocardial blood flow is also at its highest. Systole represents the low points when the heart is contracted. Chamber blood flow is low due to blood ejection. Shortening of the muscle fibres compresses the microvasculature of the myocardium and thus decreases flow. LSCI only penetrates some

thickness of the ventricular wall and hence recordings do not include chamber blood flow. **(C)** *Basic Speckle Analysis software* (SpAn), written in-house, allows us to identify and collate the high and low points from flux images. In order for points to be considered as high and low peaks, the two points prior and after them must have the correct trajectory i.e. high peaks must be followed by two decreasing flux points. If not, these peaks are disregarded (marked in white on the SpAn output). These high and low inflection points, indicated by green and blue circles, can then be collected and used for analysis. The data presented in this paper analyses the high points or flux during diastole. **(D)** Some component of the flux values derives from movement. Therefore, to ensure the readings obtained were not simply attributable to heart movement, a small thick piece of tissue was placed on the surface of the heart to prevent recordings from underlying muscle and only capture of flux due to motion of the heart. Flux readings were then taken from both the tissue and the heart and compared. The red shape on the photo and flux image represents the area analysed on heart and the blue/black an area on the tissue. The analysis graph shows that although some proportion of the flux value derives from movement, there is a remainder which appears to be attributable to the blood flow events taking place in the heart.

Supplementary Figure 3. Myocardial IRI is associated with microvascular endothelial oxidative stress. (A&B) Inflammatory damage within IRI frozen heart sections was demonstrated immunohistochemically by significantly increased VCAM-1 expression (Alexa 647-conjugated rat anti-mouse CD106 ab; BioLegend, USA). Although intense fluorescence was noted on larger coronary vessels, myocardial capillaries also supported VCAM-1 expression. **(A&C**) Significantly increased staining of 8-OHdG was also seen on injured sections (polyclonal goat anti-mouse 8-OHdG ab and Alexa 488-conjugated donkey anti-goat IgG antibody; Abcam, UK). Co-staining with CD31 demonstrated that the endothelium, particularly of larger coronary vessels, was a key target of oxidative damage. However, it was clear that a significant proportion of microvessels and capillaries also underwent oxidative stress. Image analysis was performed using ImageJ software in order to generate an integrated intensity value. **(D)** IR injured frozen heart sections (10μm) were able to significantly support HSPC adhesion when compared to sham sections. (n=3; t-test).

Supplementary Figure 4. Flow cytometry data demonstrating the perventage of HSPCs identified in the digested mouse (A) heart, (B) liver and (C) lungs following systemic administration in sham and IRI mice. Tissue was digested 2 hrs post-reperfusion and the percentage of CFSE-labelled HSPCs obtained from a total of 100,000 events quantitated. No significant differences in the number of HSPCs between sham and IRI mice was identified in any organ. Generally, fewer cells were identified within the hearts of sham or IRI mice when compared to cells present in the liver and lungs. In the latter two organs a similar mean number of cells were present. (n=3; t-test).

SUPPLEMENTARY VIDEOS

Supplementary Video 1

‘Adherent’ neutrophil within a sham beating mouse heart ‘patrolling’ within the capillary and not just standing still.

Supplementary Video 2

‘Adherent’ neutrophil within an IR injured beating mouse heart ‘patrolling’ within the capillary and not just standing still – circulating neutrophils can also be seen.

Supplementary Video 3

Adherent neutrophils (green) within a sham beating mouse heart, but circulating ones also seen indicative of good perfusion.

Supplementary Video 4

Adherent neutrophils (green) and platelet microthrombi (red) within an IR injured beating mouse heart.

Supplementary Video 5

Circulating neutrophil (green; circled) blocked by an adherent platelet microthrombus (red) occluding a capillary in an IR injured beating mouse heart.

Supplementary Video 6

CFSE-labelled HSPCs circulating through an IR injred beating mouse heart but none becoming retained.

Supplementary Video 7

Laser speckle contrast imaging of a beating mouse heart prior to induction of IR injury. The left lung is also seen.

Supplementary Video 8

Laser speckle contrast imaging of a beating mouse heart 90 minutes post-reperfusion indicating a sustained hyperaemic response. The left lung is also seen.

Figure S1

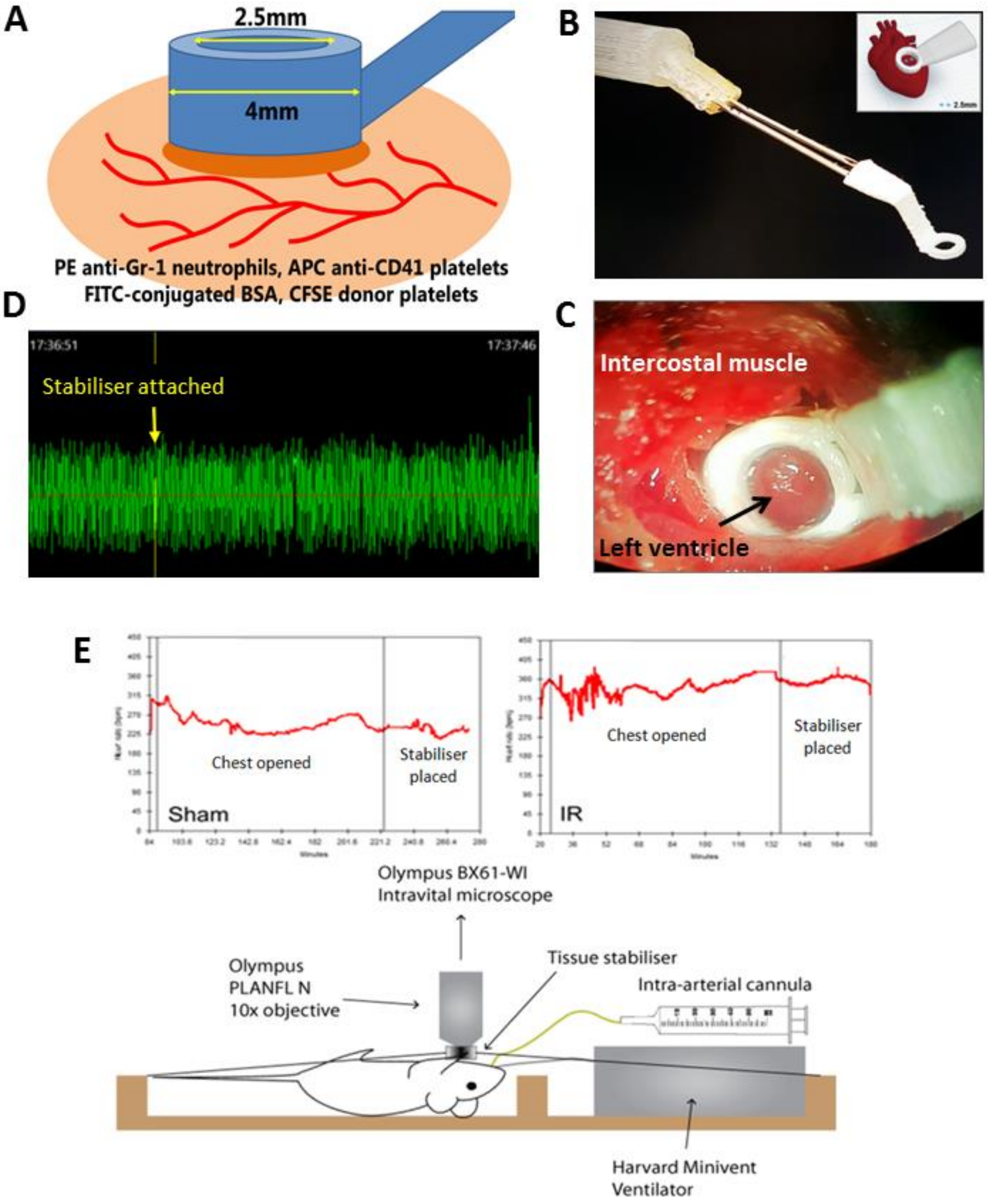
A
2.5mm
4mm
PE anti-Gr-1 neutrophils, APC anti-CD41 platelets
FITC-conjugated BSA, CFSE donor platelets
B
C
Intercostal muscle
Left ventricle
D
17:36:51
17:37:46
Stabiliser attached
E
Chest opened
Stabiliser placed
Sham
IR
Olympus BX61-WI
Intravital microscope
Olympus
PLANFL N
10x objective
Tissue stabiliser
Intra-arterial cannula
Harvard Minivent
Ventilator

Figure S2

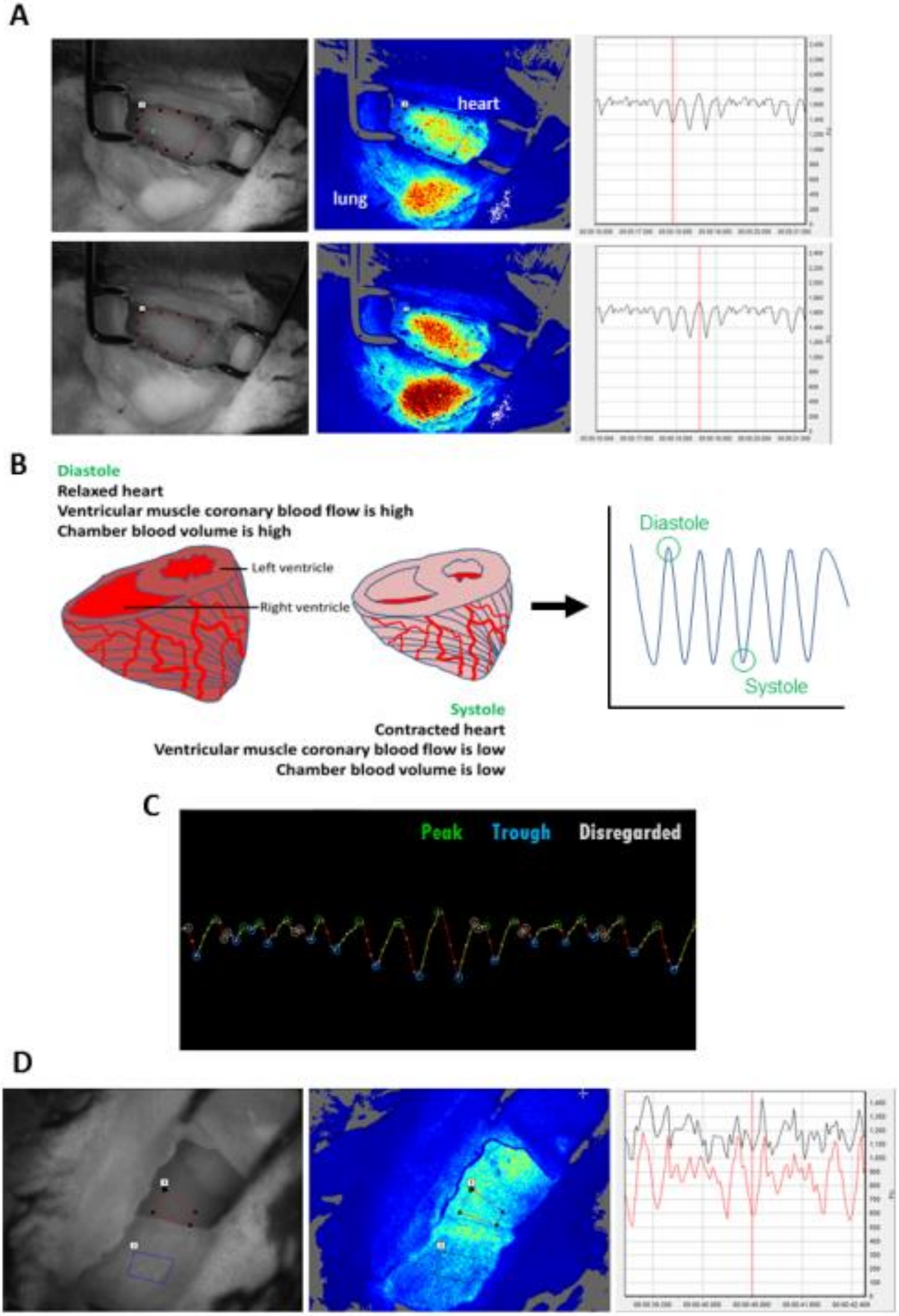

A
heart
lung
B
Diastole
Relaxed heart
Ventricular muscle coronary blood flow is high
Chamber blood volume is high
Left ventricle
Right ventricle
Diastole
Systole
Systole
Contracted heart
Ventricular muscle coronary blood flow is low
Chamber blood volume is low
C
Peak
Trough
Disregarded
D

Figure S3

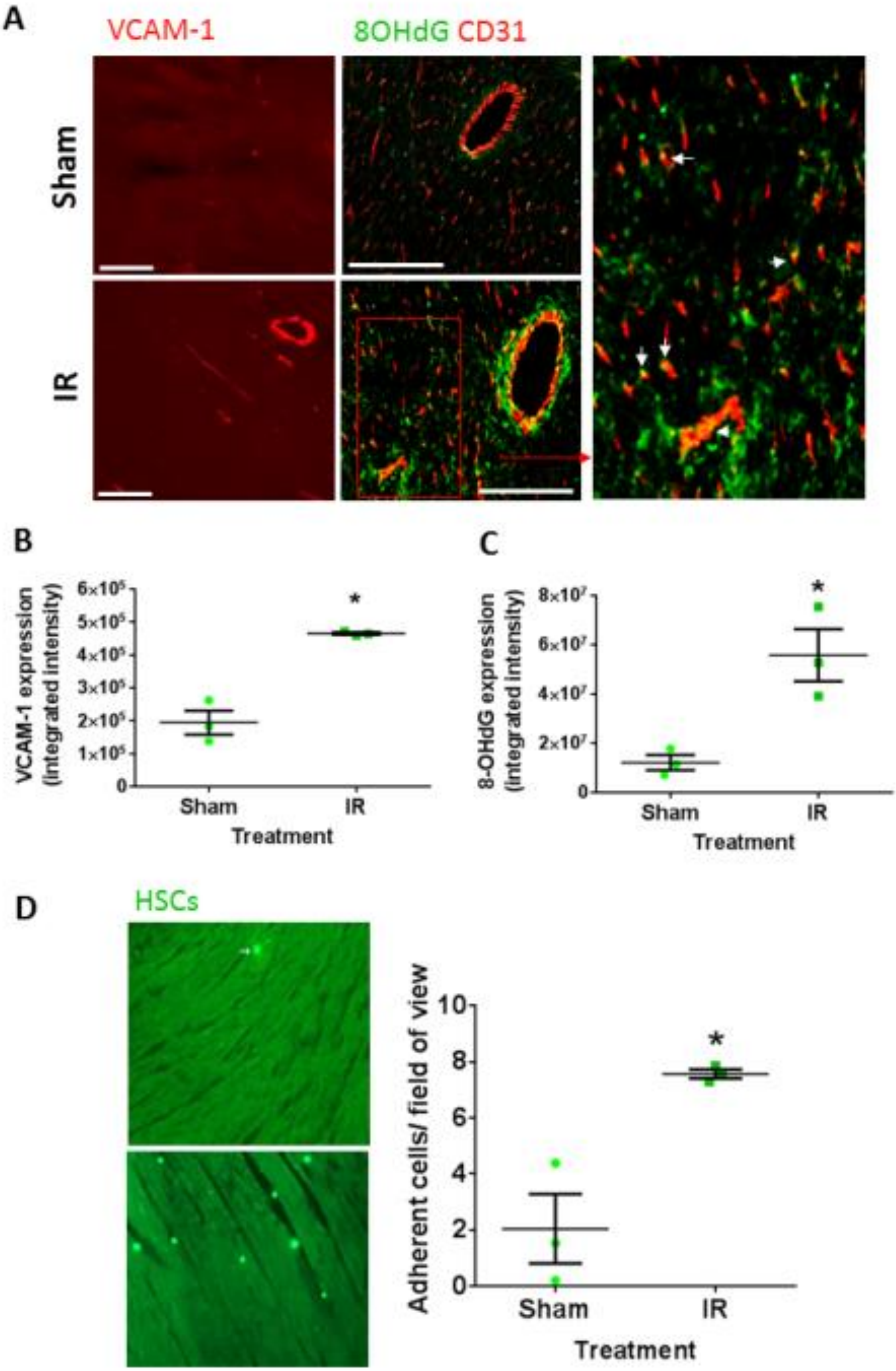
A
VCAM-1
8OHdG CD31
Sham
IR
B
6×10⁵
5×10⁵
4×10⁵
3×10⁵
2×10⁵
1×10⁵
0
VCAM-1 expression (integrated intensity)
Sham
IR
Treatment
*
C
8×10⁷
6×10⁷
4×10⁷
2×10⁷
0
8-OHdG expression (integrated intensity)
Sham
IR
Treatment
*
D
HSCs
10
8
6
4
2
0
Adherent cells/ field of view
Sham
IR
Treatment
*

Figure S4

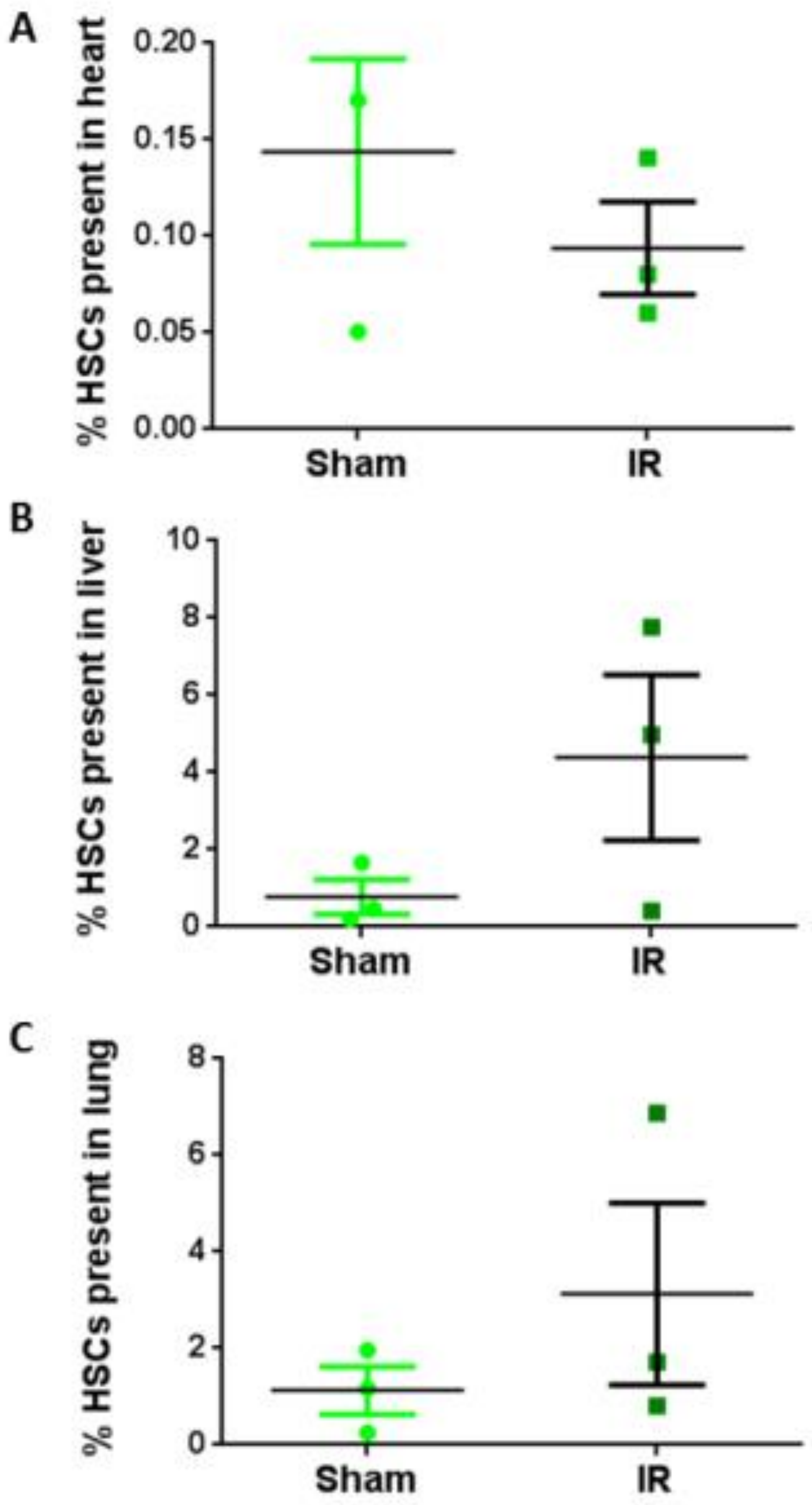
A
% HSCs present in heart
0.20
0.15
0.10
0.05
0.00
Sham
IR
B
% HSCs present in liver
10
8
6
4
2
0
Sham
IR
C
% HSCs present in lung
8
6
4
2
0
Sham
IR
